# Resilience and comfort with advance care planning among adult outpatients with congenital heart disease

**DOI:** 10.1016/j.ijcchd.2026.100690

**Published:** 2026-06-25

**Authors:** Sam L. Tsai, Lyndia C. Brumback, Ronald A. Palmen, Joyce P. Yi-Frazier, Adrienne H. Kovacs, Abby R. Rosenberg, Jill M. Steiner

**Affiliations:** a Department of Pediatrics, Seattle Children's Hospital, Seattle, WA, 98105, USA; b Department of Biostatistics, University of Washington, Seattle, WA, 98195, USA; c Department of Internal Medicine, The University of Kansas, Kansas City, KS, 66103, USA; d Dana-Farber Cancer Institute Center for Clinical and Translational Research, Boston, MA, 02215, USA; e Seattle Children's Research Institute, Seattle, WA, 98101, USA; f Department of Psychosocial Oncology and Palliative Care, Dana-Farber Cancer Institute, Harvard Medical School, 450 Brookline Ave, Boston, MA, 02215, USA; g Equilibria Psychological Health, Toronto, Ontario, Canada; h Department of Psychosocial Oncology and Palliative Care, Dana-Farber Cancer Institute, Boston, MA, 02215, USA; i Department of Pediatrics, Boston Children's Hospital, Boston, MA, 02215, USA; j Department of Pediatrics, Harvard Medical School, Boston, MA, 02215, USA; k Division of Cardiology, Department of Medicine & Cambia Palliative Care Center of Excellence, University of Washington, Seattle, WA, 98195, USA

**Keywords:** Adult congenital heart disease, Resilience, Advance care planning, Mental health

## Abstract

**Background:**

Adults with congenital heart disease (ACHD) have a lifelong health condition that may diminish quality of life (QOL) and cause serious illness. Promoting resilience and advance care planning (ACP) can both improve QOL. The relationship between resilience and patients’ comfort discussing ACP is unknown.

**Objectives:**

This study evaluates the association between patients’ self-reported resilience and comfort discussing ACP. It also assesses the association between prior clinical events and resilience and comfort discussing ACP.

**Methods:**

We conducted a cross-sectional study of outpatients with moderate or complex ACHD. Self-reported resilience and comfort discussing ACP were evaluated using surveys. Demographic and clinical data were collected from surveys and health records. We performed linear and logistic regression to evaluate relationships.

**Results:**

Among 149 patients (41 ± 14 years; 52% female), 84% were non-Hispanic White and 28% had complex ACHD. Mean resilience was 29.0 ± 7.4; 42% reported being very comfortable talking about ACP with family and friends, and 56% with their ACHD clinician. Higher resilience was associated with higher odds of feeling very comfortable discussing ACP with families and friends (OR 1.06, 95% CI 1.01–1.12, p = 0.015) and ACHD clinicians (OR 1.10, 95% CI 1.04–1.15, p < 0.001). Participants with prior mental health clinical events (therapist encounter, psychiatric medication prescription, mental health provider encounter) tended to report lower mean resilience and similar comfort discussing ACP.

**Conclusion:**

Patients who reported higher resilience were more likely to feel comfortable discussing ACP. Strengthening resilience may be an avenue for supporting patients with ACHD to more comfortably engage in ACP.

## Highlights

• Promoting resilience and advance care planning in ACHD may improve quality of life.
• There appears to be an association between resilience and comfort with ACP.
• Resilience and comfort with ACP may reflect patients' overall psychological well-being.
• Strengthening resilience may support patients with ACHD engage in ACP discussions.

## Graphical abstract

**Figure:**
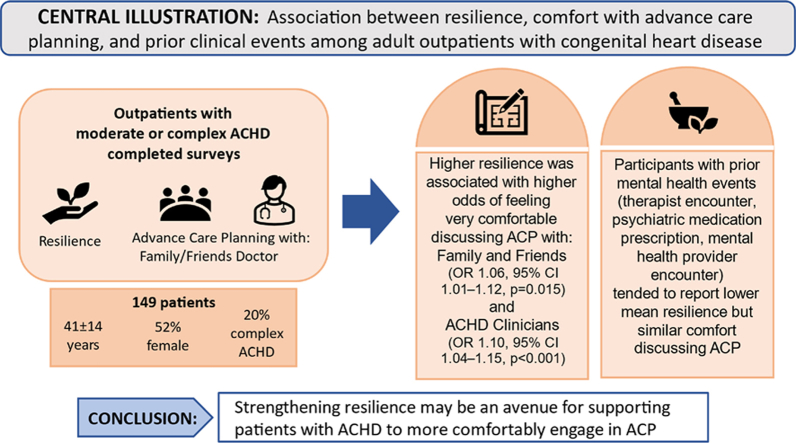

## Abbreviations list:

ACHD: adult congenital heart disease
QOL: quality of life
ACP: advance care planning
CD-RISC-10: Connor-Davidson Resilience Scale
ER: emergency room

## 1 Introduction

People living with adult congenital heart disease (ACHD) often face physical and emotional challenges in the form of health-related limitations, comorbid disease, and impaired well-being and quality of life (QOL) [[1], [2], [3]]. Indeed, feelings of vulnerability during times of deteriorating health, or uncertainty about future health, have been observed as early as in childhood [4]. Additionally, the average life expectancy across all types of ACHD, although much improved compared to prior decades, remains approximately 45-55 years [5,6]. Serious illness can occur unexpectedly and at any time, and an estimated 64%-87% of patients with ACHD die in hospital, with substantial healthcare utilization toward the end of life [[7], [8], [9]].

Given these ongoing health challenges, there is growing interest in the role of palliative care in ACHD to help patients manage serious illness [10,11]. Palliative care is a type of medical care that focuses on alleviating suffering and improving QOL [12]. Advance care planning (ACP), one component of palliative care, involves proactively discussing and preparing for future healthcare decisions [13]. Patients with ACHD generally recognize the value of ACP and are willing to participate in ACP discussions, especially with their ACHD clinicians and prior to the development of serious illness [11,14,15]. Yet despite potential benefits, ACP is rarely initiated in ACHD [11,16], and racial and ethnic disparities may exist [17]. This may partly reflect both patient and physician hesitancy, as well as unanswered questions about appropriate timing [18].

Resilience is the process of harnessing resources to sustain well-being in the face of stress [19]. Strengthened by experience or intentional practice, successful navigation of the challenges of serious illness can help alleviate psychological distress and support self-efficacy, positively altering later health outcomes [20,21]. Although minimally studied in ACHD, resilience has been associated with better QOL [22] and may therefore hold potential to assist with managing specific psychosocial experiences in this population, such as concerns surrounding the gravity of ACP conversations.

The role of prior clinical events experienced by patients also merits inclusion in attempts to understand resilience and ACP communication. For example, it is theorized that clinical events might provide opportunities to practice coping strategies and thereby strengthen resilience. They might also prompt decision-making and readiness for conversations regarding ACP [23,24]. However, in one study, medical complications were associated with lower resilience among patients with cancer [25], and in ACHD, the impact of disease severity on resilience is unclear [[22], [23], [24]].

Associations between resilience, comfort discussing ACP, and prior clinical events in adults with ACHD remain unknown. The purpose of this study was to investigate the relationships between these concepts, as this information might facilitate achieving earlier and more comprehensive ACP in ACHD. We hypothesized that (i) patients with higher resilience would have greater comfort discussing ACP with their friends and families as well as ACHD clinicians, and (ii) prior clinical events would be associated with both higher resilience and comfort discussing ACP.

## 2 Methods

### 2.1 Participant recruitment and study procedure

We conducted a cross-sectional analysis of baseline data from a prospective cohort study examining resilience in patients receiving outpatient care at a large academic ACHD care center (Central Illustration). Details of data collection have previously been published [22]. Briefly, eligible patients were at least 18 years old with confirmed moderate or severe ACHD anatomic complexity, and with class B, C, or D physiology [2], meaning at least mild disease sequelae were present. Patients with another life-limiting condition or who were unable to independently complete surveys in English were excluded.

Data were collected between May 2021 and June 2022. Eligible patients were approached in-person at an ACHD clinic appointment or contacted by phone or email. Interested patients provided informed consent, after which they completed an electronic survey. No information was collected from those who declined study participation. Up to 5 attempts at contact (in-person, phone or email) were made for participants to complete surveys. A small gift card incentive was provided at survey completion. This study was approved by the University of Washington Institutional Review Board.

### 2.2 Study measures

Resilience was measured using the Connor-Davidson Resilience Scale (CD-RISC-10), a validated 10-item assessment with questions about personal problem-solving and approaches to adversity [26]. Each item is scored on a 5-point scale (0-4), with total scores ranging from 0 to 40. Higher scores indicate higher self-perceived resilience. The mean score of a national random sample of United States adults was 32 [27]. The survey also included 2 study-specific Likert scale-based questions asking how comfortable patients felt talking about ACP. The questions were 1) “How comfortable are you talking about future health expectations with family and friends?” and 2) “How comfortable are you talking about future health expectations with your [ACHD] medical team?” Answer choices were 0 (not comfortable at all), 1 (somewhat comfortable), 2 (comfortable), and 3 (very comfortable). Participants also provided demographic data including age, gender, marital status, educational level, employment status, race, and ethnicity.

Manual electronic health record abstraction was performed to capture data on ACHD anatomic and physiological classification, and on the presence of clinical events within the year prior to survey completion. Clinical events included (i) medical encounters (heart failure diagnosis; emergency room (ER) visit; hospitalization), (ii) mental health encounters (psychology/psychiatry encounter; therapist encounter; psychiatric medication prescription), and (iii) ACP/palliative care (documentation of ACP, such as an advance directive or living will; palliative care consultation).

### 2.3 Data analysis

We evaluated the association between patients’ reported resilience and comfort discussing ACP using logistic regression. There were less than 10% missing data in the original dataset (in variables unrelated to this manuscript), and this analysis was performed using complete data only. We used resilience as the independent variable to evaluate the hypothesis that patients with higher resilience may be more comfortable discussing ACP for future health decisions. We modeled the two dependent variables, comfort discussing ACP (1: with family and friends; and 2: with their ACHD clinician) separately and as binary: “not at all, somewhat comfortable, or comfortable” versus “very comfortable.” We used this binary response to examine the extreme of comfort against other degrees of response, which suggest at least some amount of hesitation or discomfort. This was done to maximize discriminatory ability during analysis.

In addition to fitting an unadjusted model (Model A), we fit two sets of multivariate models. In Model B, we adjusted for relevant socio-demographic variables: ACHD anatomy (moderate vs complex), age, and gender (female vs male; 2 patients who answered “transgender” were excluded from regression analyses due to small numbers). In Model C, we additionally adjusted for two specific clinical events: hospitalization and mental health provider encounter (psychology/psychiatry encounter or therapist encounter).

Lastly, we assessed whether various clinical events were separately associated with 1) resilience, 2) comfort discussing ACP with family and friends, and 3) comfort discussing ACP with ACHD clinicians. The predictors of interest were (binary yes/no) and modeled separately: heart failure diagnosis, ER visit, hospitalization, documentation of ACP, palliative care consultation, psychology/psychiatry encounter, therapist encounter, and psychiatric medication prescription, and mental health provider encounter. We performed linear regression when resilience was the outcome, and logistic regression when comfort discussing ACP was the outcome. In adjusted analyses, we accounted for ACHD anatomy, age, and gender.

## 3 Results

### 3.1 Participant sample description

Of 172 enrolled patients, complete data for variables in the analysis was available for 149 participants (mean age 41 ± 14 years, 52% female; Table 1). Mean (SD) resilience was 29.0 ± 7.4. Of the final sample, 42% reported being very comfortable talking about ACP with family and friends, and 56% reported being very comfortable talking about ACP with their ACHD clinician. Proportions of participants with the various clinical events ranged from 7% (Palliative Care Consultation) to 40% (ER visit).

**Table 1:** Participant characteristics.

Characteristic	N (%) (N = 149)
Sociodemographic Characteristics	
Age (years)	
Mean (±SD)	41 (±14)
Gender	
Female	77 (52%)
Male	70 (47%)
Transgender	2 (1%)
Marital Status	
Single	41 (28%)
Married	77 (52%)
Partnered, but not married	20 (13%)
Divorced/Separated	7 (5%)
Widowed	3 (2%)
Prefer not to answer	1 (1%)
Race and Ethnicity	
White	125 (84%)
Black	4 (3%)
Asian	7 (5%)
American Indian/Alaskan Native	1 (1%)
Hispanic/Latino	6 (4%)
Other	4 (3%)
Prefer not to answer	2 (1%)
Education	
≤8th Grade	1 (1%)
High School or equivalent	40 (27%)
4-year College degree	61 (41%)
Graduate degree	34 (23%)
Other	12 (8%)
Prefer not to answer	1 (1%)
Employment	
Employed for wages	82 (55%)
Self-employed	15 (10%)
Homemaker	7 (5%)
Student	13 (9%)
Retired	12 (8%)
Out of work	6 (4%)
Unable to work/Disabled	9 (6%)
Other	4 (3%)
Prefer not to answer	1 (1%)
Insurance	
None	5 (3%)
Public	29 (20%)
Private	113 (76%)
Other	2 (1%)
ACHD Anatomy Severity	
Moderate	108 (72%)
Complex	41 (28%)
ACHD Physiology Severity	
B	84 (56%)
C	57 (38%)
D	8 (5%)
Heart Failure Diagnosis	43 (29%)
Prior Clinical Events (within 1 year)	
Hospitalization	39 (26%)
Emergency Room Visit	59 (40%)
Advance Care Planning Documentation	32 (21%)
Palliative Care Consult	10 (7%)
Psychology/Psychiatry Encounter	32 (21%)
Therapist Encounter	49 (33%)
Mental Health Provider Encounter	81 (54%)
Psychiatric Medication Prescription	56 (38%)

### 3.2 Associations between resilience and comfort with advance care planning

Resilience scores varied by comfort discussing ACP both with 1) family and friends and 2) ACHD clinicians. For family/friends, mean ± SD resilience score was 30.9 ± 7.8 for those who selected “very comfortable” versus 27.8 ± 6.9 for those less comfortable. For ACHD clinicians, mean ± SD resilience score was 31.1 ± 7.4 for those who selected “very comfortable” versus 26.5 ± 6.6 for those less comfortable. Higher resilience was associated with higher odds of feeling very comfortable discussing ACP with 1) family and friends, and 2) ACHD clinicians (Table 2). A one-point higher score on the CD-RISC-10 was associated with a 6% higher odds of being very comfortable discussing ACP with family and friends (OR 1.06, 95% CI 1.01–1.12, p = 0.015) and a 10% higher odds of being very comfortable discussing ACP with ACHD clinicians (OR 1.10, 95% CI 1.04–1.15, p < 0.001). Results were similar after adjustment for relevant sociodemographic variables and clinical events (Models B and C, Table 2).

**Table 2:** Associations between resilience and comfort with advance care planning.

	Odds Ratio	95% Confidence Interval	P-value
Model A (Unadjusted)			
Comfort Family/Friends	1.06	1.01–1.11	0.02
Comfort ACHD Clinician	1.10	1.04–1.15	<0.001
Model B (Adjusted for sociodemographic variables)			
Comfort Family/Friends	1.06	1.01–1.12	0.01
Comfort ACHD Clinician	1.11	1.05–1.17	<0.001
Model C (Additionally adjusted for prior clinical events)			
Comfort Family/Friends	1.07	1.02–1.13	0.01
Comfort ACHD Clinician	1.12	1.06–1.18	<0.001

### 3.3 Associations with prior clinical events

Prior clinical events were associated with lower mean resilience. The difference in mean resilience was −3.84 (95% CI -6.79 to −0.89, p = 0.01) for having had a prior psychology/psychiatry encounter, −4.63 (95% CI -7.12 to −2.14, p=<0.001) for therapist encounter, −3.02 (95% CI -5.48 to −0.56, p = 0.02) for psychiatric medication prescription, and −4.59 (95% CI -7.01 to −2.18, p=<0.001) for “mental health provider encounter.” Results were similar in adjusted analyses. None of the prior clinical events were significantly associated with comfort in discussing ACP.

## 4 Discussion

That ACP is under-utilized in ACHD has been demonstrated, however factors influencing this have not been extensively investigated, nor has the development of possible solutions to encourage uptake. The primary finding of this study was that patients who reported higher resilience were more likely to report feeling comfortable discussing ACP, both with their family and friends and with their ACHD clinician. Perhaps higher resilience suggests they feel more able to effectively cope with current ACHD-related stressors, and thus feel more open to discussing future health expectations. Similarly, in caregivers of patients with advanced cancer, higher resilience has been associated with lower anxiety and depression symptoms and higher readiness for surrogate decision-making, which may promote engagement in ACP [28]. Alternatively, it is possible that patients who are more comfortable with ACP report higher resilience, given the cross-sectional nature of this study. Patients who have previously engaged in ACP may feel less uncertainty about the future, or more in control of their condition and healthcare [13,29], so may perceive themselves as more resilient. However, this seems less likely than the former suggestion, given the generally low levels of ACP in general in this population and the low proportion of patients with ACP documentation in the electronic health record or prior palliative care consultation in this study.

Having previously received mental health treatment (psychology/psychiatry consultation, therapist/mental health provider encounter, or having been prescribed a psychiatric medication) were all associated with lower mean resilience in this study. This finding likely reflects impaired psychological well-being, with higher baseline psychological distress rather than truly reduced resilience. Indeed, high levels of anxiety and depression have been reported in the ACHD population across multiple studies [3,30]. In this cross-sectional study, it is important to remember that directionality and timing of associations is unclear, and causal inferences cannot be made. People with higher distress may have appropriately received professional support; perhaps their sense of resilience has yet to improve. Nevertheless, impaired psychological well-being may reduce patients’ ability to plan for future healthcare decisions through ACP. Thus, understanding relationships between current resilience and participation in ACP and other healthcare discussions is key.

Interestingly, this study demonstrates an absence of associations between prior clinical events and improved ACP comfort. A widely held belief is that exposure - having already faced difficult medical decisions - might increase comfort. However, there could be many reasons to explain this finding. Importantly, not all clinical experiences may build patients’ confidence in handling difficult conversations like planning for the future; it is possible that exposure could yield feelings of worse uncertainty or control. Additionally, the degree of clinical change may be more subtle in the ACHD population, as they are reminded of expected challenges and anticipated changes throughout their lives, and they may not interpret changes in diagnosis or function in the same way as clinicians who have the advantage of observing outcomes for dozens of patients.

There are several potential implications from this study. That there seems to be an association between resilience and ACP comfort suggests that strengthening resilience may support patients with ACHD to more confidently engage in ACP conversations. The application of behavioral interventions designed to enhance resilience is one way to accomplish this goal and should be examined in this population. Early trials are promising [31,32] - that interventions seem feasible and well-received by patients, although large efficacy studies have not yet been performed. A major unanswered question is what is the ideal timing and patient who may most benefit from such an intervention. In clinical practice, these topics may be addressed through dedicated psychological counseling or treatment, employing techniques like cognitive behavioral therapy and acceptance and commitment therapy. Resilience and comfort discussing ACP may be a reflection of patients' overall psychological well-being. In our study, patients who had received some mental health treatment had lower resilience on average, and patients who had lower resilience felt less comfortable discussing ACP on average. Strengthening the psychological well-being of these patients, who are recognized to experience high rates of anxiety and depression [31], is especially important. In a previous study, Deng et al. demonstrated that patients with more anxiety symptoms assigned greater importance to ACP [29]. Encouraging ACP may decrease patients’ uncertainty, and therefore anxiety, about the future. Expanding awareness of ACP through regular, normalized discussions with trusted ACHD clinicians may decrease the emotional weight of this topic, allowing for stronger feelings of control and increased comfort among patients.

This study has several limitations. Despite this study being conducted in a large subspecialty ACHD care center, the sample size was relatively small. Over half of the participants had education beyond high school, race and ethnicity data were included in the survey as one variable, most participants were non-Hispanic White, and (due to small numbers), 2 patients who reported being transgender were excluded from analyses. These characteristics raise the question of possible recruitment bias, and limited generalizability. However, the study size is comparable to many studies involving the ACHD population, particularly those investigating psychosocial health, and, as this question has not previously been investigated, it provides important hypothesis-generating data. Additionally, the questions about ACP comfort were study-specific and not formally validated. These items were designed to use lay language easily understood by participants in the context of the overall study. Patients were not specifically asked about ACP-related decision-making, rather the concept of ACP discussions as a way to prepare for future healthcare decisions. Psychosocial concerns like self-perceived resilience and patients’ comfort with ACP discussions require self-reporting and are best described through patient-reported outcomes measures, as employed in this study. Finally, this was a cross-sectional analysis [22]. There may be unmeasured confounders, and causal relationships may not be concluded. Future longitudinal, multicenter studies of resilience and ACP should be conducted to better understand these relationships.

In summary, this study suggests associations between patients' self-perceived resilience, comfort discussing ACP, and the impact of prior clinical events, in particular patients’ mental health treatment history. Knowledge of these associations may help ACHD clinicians develop strategies to encourage earlier and more comprehensive ACP. Doing so may lead to improved psychological well-being and QOL outcomes, and perhaps ensure more efficient and appropriate healthcare utilization.

## Clinical perspectives

Clinical Implications.

• Clinician awareness of these associations can assist with conversations about mental health
• Familiarity with aspects of resilience may help achieve earlier and more comprehensive advance care planning

Translational Outlook.

• Larger and longitudinal studies should further explore the relationship between resilience, advance care planning, and clinical events

## Funding

Dr. Steiner is supported by a career development award from the 10.13039/100000002National Institutes of Health, K23HL15180.

## CRediT authorship contribution statement

**Sam L. Tsai:** Conceptualization, Data curation, Formal analysis, Investigation, Project administration, Writing – original draft. **Lyndia C. Brumback:** Data curation, Formal analysis, Methodology. **Ronald A. Palmen:** Formal analysis, Writing – original draft, Writing – review & editing. **Joyce P. Yi-Frazier:** Formal analysis, Methodology, Writing – review & editing. **Adrienne H. Kovacs:** Investigation, Methodology, Writing – review & editing. **Abby R. Rosenberg:** Data curation, Methodology, Writing – review & editing. **Jill M. Steiner:** Conceptualization, Formal analysis, Funding acquisition, Investigation, Methodology, Project administration, Resources, Supervision, Validation, Writing – original draft, Writing – review & editing.

## Declaration of competing interest

The authors declare that they have no known competing financial interests or personal relationships that could have appeared to influence the work reported in this paper.
